# Reproductive Characteristics of Thawed Stallion Sperm

**DOI:** 10.3390/ani9121099

**Published:** 2019-12-09

**Authors:** Mikhail M. Atroshchenko, Ekaterina Arkhangelskaya, Dmitry A. Isaev, Sergey B. Stavitsky, Alexander M. Zaitsev, Valery V. Kalaschnikov, Sergey Leonov, Andreyan N. Osipov

**Affiliations:** 1 All-Russian Scientific Research Institute for Horse Breeding (ARRIH), Ryazan Oblast, Rybnovskij District 391105, Russia; atromiks-77@mail.ru (M.M.A.); amzaitceff@mail.ru (A.M.Z.); vniik08@mail.ru (V.V.K.); 2 State Research Center—Burnasyan Federal Medical Biophysical Center of Federal Medical Biological Agency (SRC-FMBC), 46, Zhivopisnaya Str, Moscow 123098, Russia; ek.arkhangelskaya@gmail.com (E.A.); embryology@yandex.com (D.A.I.); 3 Semenov Institute of Chemical Physics, Russian Academy of Sciences, Moscow 119991, Russia; 4 Center for Veterinary Cell Medicine, 8/1 Tvardovsky Str, Moscow 123458, Russia; vcmcinfo@gmail.com; 5 Moscow Institute of Physics and Technology, Dolgoprudny, Moscow Region 141700, Russia; leonov.sv@phystech.edu; 6 Institute of Cell Biophysics, Russian Academy of Sciences, Pushchino, Moscow Region 142290, Russia

**Keywords:** horse breeding, sperm, cryopreservation, reproductive characteristics, DNA fragmentation

## Abstract

**Simple Summary:**

It is very important to evaluate correctly the fertilizing capacity of frozen sperm before carrying out the artificial insemination of mares. It is not always possible to perform complex laboratory tests under the stud farm’s conditions. The purpose of our study was to determine the minimum set of relatively simple laboratory tests sufficient to confirm the feasibility of frozen/thawed sperm for insemination and to predict the pregnancy rate in mares. We found that sperm characteristics such as activity, survival at low temperature, and DNA damage are sufficient to determine the quality of frozen/thawed sperm. These characteristics can be determined by simple tests and assays that can be used for increasing the effectiveness of artificial insemination not only in horse breeding but also in other areas of livestock and even in reproductive medicine.

**Abstract:**

The main goal of our study was to determine a set of thawed stallion sperm characteristics that have predictive value for the pregnancy rate (PR) of mares after artificial insemination (AI). DNA fragmentation and survival of sperm during hypothermic storage were studied in addition to routinely determined semen characteristics such as concentration, percentage of motile spermatozoa, and morphology. To estimate DNA fragmentation, a modified hallo assay was applied. Sperm survival was determined within hours as the ability of spermatozoa to maintain progressive motility (PM) during the storage of ejaculate diluted with lactose-chelate-citrate-yolk (LCCY) medium at +4 °C. Strong positive correlation between PR and thawed sperm motility (*r* = 0.90, *p* < 0.05) as well as between PR and sperm survival (*r* = 084, *p* < 0.05) was revealed. There was also a strong negative correlation between PR and DNA damages in spermatozoa (*r* = −0.94, *p* < 0.05). We found no dependence of PR on normal morphology spermatozoa percentage in thawed semen. We concluded that the sperm activity, survival, and DNA fragmentation should be considered as the sufficient reproductive characteristics of semen to evaluate the quality of frozen/thawed sperm and prediction of PR.

## 1. Introduction

The effectiveness of artificial insemination (AI) in animals and humans depends mainly on the sperm fertilizing capacity. A set of routine studies such as measuring the ejaculate volume and concentration, calculating the percentage of spermatozoa with progressive motility, and calculating the percentage of live and morphologically normal spermatozoa allow only an indirect estimation of sperm fertility [1,2,3]. In a number of cases, such an assessment is not sufficient to determine the possible causes of fecundity decline, in particular when cryopreserved sperm is used for the AI.

One of the main goals of pedigree horse breeding is to extend the reproduction of livestock using the most valuable genotypes of stallions. For this purpose, a regular cryopreservation of sperm is carried out. Often, the decline in sperm fertility after thawing (defrosting) remains unclear given acceptable and conventional sperm characteristics. In reproductive medicine, a number of additional laboratory tests have been recommended including functional tests for the integrity of membranes, binding of spermatozoa with hyaluronic acid, and detection of sperm DNA damages [4]. Indeed, in the practice of stud farms, additional studies of stallion sperm should include a minimum set of tests that provide an adequate estimation of sperm fertility by indirect characteristics, which are especially important for cryopreserved and thawed sperm. Therefore, the purpose of our study was to examine the prognostic value of the general characteristics, and some additional ones, of thawed stallion sperm for the pregnancy rate (PR), in order to determine a minimal set of relatively simple tests sufficient for the estimation of sperm fertility.

## 2. Materials and Methods 

### 2.1. Fresh and Frozen Stallion Sperm Samples 

The study was performed on the livestock of The All-Russian Research Institute for Horse Breeding (ARRIH, Ryazan Region, Russia) and the Tersk Stud Farm N169 (Stavropol Region, Russia). The mares and stallions were of Arabian breed. All procedures were carried out in accordance with the “European Convention for the protection of vertebrates used for experimental and other scientific purposes” ETS No. 123 (18 March 1986) and the Law of the Russia Federation on Veterinary Medicine No. 4979-1 (14 May 1993).

Samples of fresh (from 21 stallions) and cryopreserved (from 17 stallions) sperm were taken for analysis. The fresh sperm samples from stallions were obtained using an artificial vagina at intervals of 48 h. After a sexual rest period (10 days and more) of the stallions, the first two ejaculates were not used in the studies. All frozen sperm samples were obtained from the bioresource collection “Cryobank of Genetic Resources” of the ARRIH. For this study, 20 mL of sperm diluted at a ratio of 1:3 (*v*/*v*) with lactose-chelate-citrate-yolk (LCCY) medium containing 3.5% glycerin in aluminum tubes were frozen according to the standard technology (standard operating procedure (SOP)) of the All-Russian Research Institute of Horse Breeding in liquid nitrogen vapor, and the distance to the surface of liquid nitrogen was 20 mm [5]. Briefly, the polyurethane rack containing the tubes with sperm was cooled from +4 °C down to −127 °C for 420 s under the freezing rate of 3.2 °C per second. The frozen sperm samples were stored in liquid nitrogen.

#### Thawing of Frozen Sperm 

The frozen sperm was quickly thawed in a water bath at +40 °C for 60 s. The spermatozoa concentration was counted using a Goryaev’s hemocytometer. The concentration of spermatozoa in frozen semen was 45–50 million/mL.

### 2.2. Sperm Examination 

The volume of collected ejaculate was measured and the spermatozoa concentration was counted using a Goryaev’s hemocytometer. A computer-assisted sperm analysis (CASA) was used to evaluate the spermatozoa progressive motility (PM) in our study. The assessment of PM was implemented using an Argus CASA system (ArgusSoft LTD., St. Petersburg, Russia) and a Motic BA 410 microscope (Motic, Hong Kong, China) in a Mackler chamber at 37 °C. Morphological assessment of fresh and thawed sperm samples was performed using May–Grunwald–Giemsa stained smears. The morphology of at least 250 spermatozoa was examined on each slide at 1000× magnification.

#### 2.2.1. Sperm Survival Test

Once obtained, the ejaculates were diluted with lactose-chelate-citrate-yolk (LCCY) medium containing 3.5% glycerin at a ratio of 1:3 (*v*/*v*) and placed at +4 °C. Time-dependent sperm survival was determined as the ability of spermatozoa to maintain PM above 5% during the hypothermic (+4 °C) storage of diluted ejaculate. 

#### 2.2.2. Sperm DNA Fragmentation Test

Sperm DNA fragmentation was assessed using a halo assay [6,7] according to our modified procedure. Briefly, 25 μL of fresh or thawed diluted sperm (~1 million/mL in phosphate buffered saline (PBS, pH 7.4)) were mixed with 50 μL of 2% low melting agarose at +37 °C. The mixture was then placed on a slide and covered with a slip until the agarose gelled, and the cover slip was removed followed by treatment of the slide for 20 min in an alkaline solution composed of 300 mM NaOH, 1 mM EDTA, and 1% SDS. Under these conditions, fragments of damaged DNA migrate through the agarose gel. As a result, spermatozoa with damaged DNA have a colored halo around the head in the Wright-Giemsa stained slides. The resulting interpretation was confirmed by hydrogen peroxide positive control test. Treatment of diluted sperm (~1 million/mL in PBS) with 1 mM hydrogen peroxide (15 min, +4 °C) caused an approximate 2-fold increase in percentage of spermatozoa with halo. DNA fragmentation was determined as a percentage of sperm with halo; at least 250 spermatozoa were examined in each slide at 400× magnification.

### 2.3. Artificial Insemination (AI) and Pregnancy Control

One ejaculate of each stallion was used in all sperm and artificial insemination studies of the mares. AIs were performed by transcervical injection of the dose of either fresh or thawed sperm with preliminary control of follicle development 1–4 h before and after ovulation [8]. In all, 252 mares were artificially inseminated in one sex cycle. The age of the mares ranged from 5 to 12 years, and all mares gave birth earlier. Of the mares, 157 were inseminated with fresh sperm, and 95, with thawed sperm. The dose for insemination with fresh sperm contained at least 500 million spermatozoa having PM. The dose for the insemination of frozen sperm was 250 million spermatozoa with PM. Possible sperm retention and postcoital endometritis were evaluated by a mandatory ultrasound examination of the uterus within 24 h after artificial insemination and ovulation of mares. Pregnancy was determined by ultrasound examination (from the 14th day after ovulation) and by rectal palpation (from the 18th day after ovulation). The number of pregnant mares was counted in each AI cycle.

### 2.4. Statistical Analysis

We used the non-parametric Mann–Whitney *U* test in our statistical evaluation of the data of ejaculate samples from different stallions. Spearman’s rank correlation was used to reveal the strength of a link between data sets. Data are presented as M ± SEM, where M is the mean and SE is the standard error of the mean.

## 3. Results

Instead of the AI of mares with fresh sperm, acute pregnancy was achieved by live cover, so that all the sperm assays were made for previously obtained ejaculates. For this reason, the study of correlations between PR and the characteristics of fresh sperm was not carried out. Nevertheless, it should be noted that the average PR per cycle of natural covering was as high as 81.3 ± 2.7%. For AI with thawed sperm, the average PR per cycle was significantly lower at 47.3 ± 10%. 

Morphological characteristics of the fresh and thawed semen did not differ significantly (Table 1). There was no correlation between the percentage of morphologically normal spermatozoa and PR.

**Figure 1:**
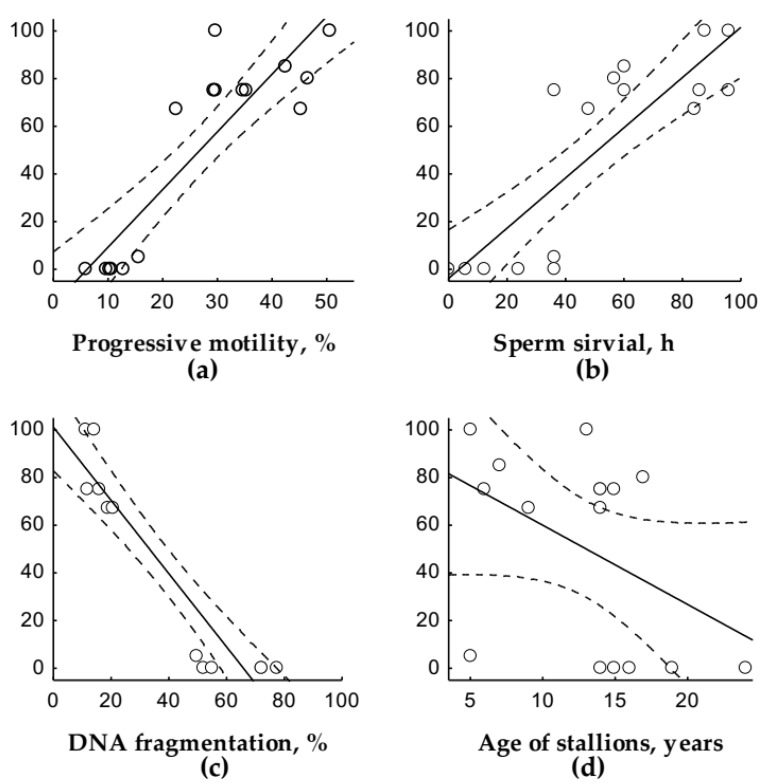
Correlation analysis of the pregnancy rate (PR) and certain characteristics of thawed sperm. (**a*,*b**) Very strong positive correlation with sperm activity (*r* = 0.90; *p* < 0.00) and sperm survival (*r* = 0.84; *p* < 0.00), respectively; (**c**) very strong negative correlation with DNA fragmentation in spermatozoa (*r* = −0.94; *p* < 0.00); and (**d**) moderate negative correlation with age of stallions at the time of sperm freezing (*r* = −0.51; *p* = 0.04). The *y*-axis indicates PR, %. The dotted lines show the 95 % confidence intervals.

The activity of the thawed sperm as well as its survival were significantly decreased compared to fresh sperm (Table 1). The strong positive correlation between activity and survival was demonstrated for the thawed sperm (*r* = 0.87; *p* < 0.00), and this dependence was less strong (*r* = 0.63; *p* < 0.00) for the fresh one. The strong positive correlations between PR and activity as well as between PR and survival of thawed sperm were revealed (Figure 1a,b).

In the case of thawed sperm, a very strong negative correlation between DNA fragmentation and its activity (*r* = −0.92; *p <* 0.00) as well as its survival (*r* = −0.95; *p* < 0.00) was found. For the fresh sperm, DNA fragmentation was also negatively correlated with its activity (*r* = −0.65; *p* < 0.00), although the correlation between DNA fragmentation and sperm survival was not revealed in contrast to thawed sperm. A very strong negative correlation was found between DNA fragmentation and PR (Figure 1c).

Depending on the stallion age, there was also a decrease in activity (*r* = −0.54; *p* = 0.03) and survival (*r* = −0.58; *p* = 0.02) of thawed sperm, although there was no correlation between PR and the age of stallions after live cover. For AI with thawed sperm, the correlation was moderately negative (Figure 1d). 

The correlation between the age of the stallions and DNA fragmentation in thawed sperm was positive (*r* = 0.64; *p* = 0.03). In contrast, there was no such correlation for fresh sperm (*p* = 0.34). 

## 4. Discussion

DNA fragmentation in spermatozoa can dramatically affect fertility. Morrel et al. pointed out the significant negative correlation between the sperm DNA fragmentation index and fertilization rate in horse [2]. Some authors considered nuclear chromatin integrity as a marker of sperm quality that is used to determine its reproductive capability in farm animals and humans [9,10,11,12]. In our study, the sperm DNA fragmentation of both fresh and thawed sperm was rather high (Table 1). According to López-Fernández et al., the DNA fragmentation indices both in fresh stallion sperm instantly after collection and in thawed sperm immediately after thawing did not differ and were about 15%; but after the hypothermic storage for an hour, DNA fragmentation increased by more than 30% [13], which is similar to our data. Krakowski et al. suggested that the evaluation of membrane integrity and DNA fragmentation could be useful as an additional predictive criteria for assessing the quality of stallion sperm when stored at +4 °C [14].

Giannoccaro et al. noted a weak correlation between DNA fragmentation in stallion spermatozoa and their mobility and viability [15]. At the same time, other authors reported a negative effect of chromatin damage on the motility of human spermatozoa [16,17] and their fertilizing ability [1]. Our data support these observations, although the correlation between DNA fragmentation and either sperm motility or survival was more pronounced for thawed sperm than for the fresh one.

## 5. Conclusions

Our study demonstrated the predictive value of DNA fragmentation and sperm survival in the hypothermic state for PR as well as routinely examined sperm activity. At the same time, sperm morphology and the age of stallions may have less predictive PR value, perhaps due to the selection for cryopreservation of sperm samples with expectedly better characteristics from both fecund stallions and those that were not too old. In general, the obtained data strongly support the notion that the fertility of stallion thawed sperm can be estimated by a limited set of characteristics, where the activity, survival, and sperm DNA fragmentation have the most prognostic PR value. 

## Figures and Tables

**Table 1:** Characteristics of fresh and thawed stallion sperm.

Age of Stallions, Years	Sperm Volume, mL	Semen Concentration, 10^6^ per mL	Spermatozoa of Normal Morphology, %	Progressive Motility, %	Sperm Survival, hours	Spermatozoa with Damaged DNA, %
Fresh sperm (*n* = 21)						
11.2 ± 1.1	42.3 ± 5.0	210.7 ± 22.1	74.6 ± 2.5	50.7 ± 1.7	117.7 ± 9.2	28.7 ± 2.1
Thawed sperm (*n* = 17)						
12.9 ± 1.3	—	—	76.0 ± 2.2	26.9 ± 3.6 *	48.5 ± 8.1 *	39.3 ± 7.6

The means ± SEM are presented. * Differences are significant for *p* < 0.05.
